# IMU Error Propagation in Position Reconstruction and Its Mitigation Through Task-Informed Dual-IMU Constrained Optimization: Application to Golf Putting

**DOI:** 10.3390/s26134092

**Published:** 2026-06-27

**Authors:** Rachele Rossanigo, Diletta Balta, Niccolò Tortarolo, Marco Caruso

**Affiliations:** Department of Electronics and Telecommunications, Politecnico di Torino, 10129 Torino, Italy; rachele.rossanigo@polito.it (R.R.); diletta.balta@polito.it (D.B.); s302633@studenti.polito.it (N.T.)

**Keywords:** inertial measurement units, golf putting, trajectory reconstruction, sensor fusion, orientation estimation, drift reduction, constrained optimization, IMU double integration, kinematic constraints, IMU drift

## Abstract

**Highlights:**

**What are the main findings?**

- A constrained redundant optimization pipeline was developed to reduce velocity and position reconstruction errors in IMU-based golf putting analysis, by exploiting shared information from two IMUs mounted on the same putter and targeting the main error sources that propagate through orientation estimation, gravity removal, and double integration.
- Compared with the best tuned standard IMU pipeline for position reconstruction, the proposed method reduced the median RMS velocity norm error from 16.5 to 9.9 cm/s (about 40%) and the median RMS position norm error from 23.2 to 8.5 cm (about 63%).

**What are the implications of the main findings?**

- Combining redundant IMU information with kinematic constraints can improve trajectory reconstruction beyond fixed calibration and predefined sensor fusion tuning.
- The proposed modular framework can be extended beyond golf to other IMU-based applications requiring position reconstruction, and can be enriched with additional task specific information.

**Abstract:**

Inertial measurement units (IMUs) are promising low-cost tools for golf putting analysis, but accurate putter trajectory reconstruction remains limited by sensor errors, sensor fusion tuning, and drift accumulation during numerical integration. This study aimed to analyze error propagation in a standard IMU-based reconstruction pipeline, in which orientation is estimated to remove gravity from the accelerometer signal, followed by double integration to obtain velocity and position. In addition, a constrained redundant optimization pipeline (CROP) is proposed to improve reconstruction accuracy. CROP exploits the redundant information provided by two IMUs mounted on the same rigid body, together with task-informed kinematic constraints, to optimize residual accelerometer and gyroscope bias compensation terms and sensor fusion parameters for each recording. An amateur golfer performed 23 putting strokes with two IMUs mounted on the putter shaft and head, respectively, while a stereophotogrammetric system served as a reference. Results were compared with the standard pipeline tuned with different parameter configurations. Overall, compared with the best parameter configuration of the standard pipeline, CROP reduced the median RMS error of the velocity and position norms by about 40%, from 16.5 to 9.9 cm/s, and 63%, from 23.2 to 8.5 cm, respectively. These findings indicate reduced drift and improved trajectory reconstruction, although further improvements are required for accurate field use.

## 1. Introduction

**Figure 1:**
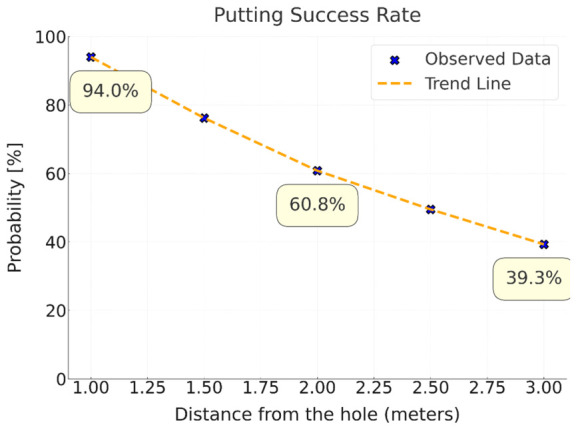
Percentage of successful putts on the PGA Tour (Professional Golfers’ Association). Adapted from [7,8].

Golf is widely recognized as a difficult sport to learn, as successful performance depends on the precise coordination of body movements with the golf club to produce the intended stroke [1,2]. A standard round is typically played over 18 holes, each ending on the putting green, where the final strokes are executed with the putter, one of the 14 clubs allowed. Although putting is often perceived as one of the simplest components of the game, it plays a substantial role in overall scoring, accounting for approximately 40% of total strokes in professional golf [3], and an even larger fraction in amateur play. Its importance is further emphasized by the sharp increase in task difficulty with distance: while professional golfers hole about 94% of putts from 1 m, success drops dramatically to about 61% from 2 m [4], as graphically shown in Figure 1. As putting distance increases, successful performance relies on increasingly precise regulation of both direction and speed under changing perceptual and mechanical constraints. Parameters such as putter face orientation and club trajectory play a key role in determining launch direction, distance control, and ball roll quality. Since less skilled golfers generally exhibit higher variability and lower precision in these kinematic features than elite players [5,6], accurate reconstruction of putter motion is essential to identify the biomechanical sources of error, quantify stroke consistency, and support targeted performance improvement through objective feedback.

In current commercial practice, putter tracking mainly relies on three technological approaches: 3D ultrasonic tracking, high speed optical tracking, and radar- or camera-based photometric imaging. For instance, SAM PuttLab is a highly accurate ultrasonic commercial system that provides more than 70 putting parameters, uses ultrasonic 3D measurement, and operates through a dedicated receiver unit connected to a PC-based interface [9]. However, it still requires dedicated hardware, calibration procedures, computer-based analysis, and an appropriate training space for reliable operation. Optical systems such as Quintic Ball Roll rely on infrared illumination and high-speed cameras, up to 1080 fps, to track both the putter and the ball throughout the impact zone without attaching devices to either the player or the club, thereby preserving natural stroke execution [10]. However, these systems remain constrained by a structured camera-based setup, the need for a workstation, dedicated space, and limited portability. Similarly, launch monitor platforms such as Trackman 4 and Foresight GCQuad provide rich club and ball data in both indoor and outdoor environments through radar and camera-based technologies. TrackMan 4 combines dual radar systems with radar-synchronized high-speed optics and provides club, ball, and putting data, while GCQuad uses quadrascopic camera-based imaging with integrated infrared light and provides putting analysis and club data [11,12]. Nevertheless, their widespread use remains limited by the cost (>10 k€ for [13]) and setup requirements typical of professional-grade launch monitor systems, rather than by the need to instrument the club directly. Overall, despite their accuracy and analytical depth, current commercial solutions remain only partially suitable for portable, low-cost, and ecologically valid putting analysis.

An alternative to these technologies is represented by inertial measurement units (IMUs). Compared with the aforementioned technologies, IMUs offer a more portable and accessible solution for putter tracking, as they are compact, battery powered, and suitable for mobile and field-based use, not requiring dedicated external equipment. Similarly to optical systems, in which the applied markers typically weigh only a few grams each, inertial sensing also requires that any device mounted on the club does not alter swing feeling. In this respect, Kawano et al. [14] showed that IMU-based sensing is feasible for putting, provided that the device remains sufficiently light, approximately 10 g.

The main limitation of IMU-based technology is that accurate trajectory reconstruction of a rigid body remains an intrinsically challenging problem in sport motion analysis, including golf [15]. The standard reconstruction pipeline consists of estimating sensor orientation, removing the gravity component from the measured acceleration, and then double integrating the resulting linear acceleration, given initial velocity conditions, to compute position. This procedure is highly sensitive to multiple sources of error, including sensor calibration inaccuracies, bias instability, orientation errors, and uncertainty in the initial conditions. Since the overall estimate effectively relies on three numerical integrations, one for orientation and two for position, even small errors can rapidly accumulate and produce position drifts ranging from several centimeters to meters over short integration intervals. Furthermore, IMU-based methods generally require task- and speed-specific tuning [16,17,18], since parameter settings that are effective for one motor task or movement intensity may not generalize to others.

This technical difficulty may explain why, in golf applications, IMUs have been adopted more often for stroke detection, phase segmentation, and temporal parameter extraction than for full club trajectory reconstruction [4,19,20]. In the specific case of putting, Jensen et al. proposed a mobile IMU-based system focused on automatic putt detection and real-time parameter computation, even though linear displacements were explicitly omitted to avoid drift and error accumulation [19]. In the limited number of studies addressing putter trajectory reconstruction, the available evidence has generally been obtained under highly controlled conditions. King et al. reported 3D club head errors of 3 mm in position and 0.5 deg in orientation, but these results were obtained with a specific setup in which the dynamic validation was performed using a single degree of freedom pendulum robot [21]. Burchfield and Venkatesan [22] bypassed the double integration step by constraining the reconstructed club motion to a parabolic path profile consistent with the motion imposed by a mechanical pendulum. This allowed the estimation to be driven by a known kinematic prior rather than by unconstrained trajectory reconstruction, yielding mean position errors of 1.6 to 6.9 mm and mean angle errors below 0.5 deg. However, such a strong assumption is also a limitation, since real putting does not follow a fixed or athlete-independent trajectory [23].

The accurate reconstruction of club-related trajectories from IMU data remains an open issue. This is also reflected by the limited number of recent studies specifically addressing full 3D club or club-related trajectory reconstruction from IMU data. In the study by Kim et al. [24], although focused on the full golf swing rather than on putting, a single wrist-worn IMU was combined with a convolutional neural network and additional kinematic constraints to reduce integration drift. Specifically, wrist velocity was assumed to be null at address, backswing top, and finish, while wrist displacement was constrained to lie on a virtual circle defined on the 3D swing plane. As in Burchfield and Venkatesan [22], however, these constraints may not be rigorously satisfied in every athlete or trial and, if not experimentally met, may themselves introduce errors that propagate throughout the pipeline. Despite the deep learning-based approach, the reported errors remained about 17 cm for trajectory estimation and about 9 deg for orientation. It should also be noted that the dynamics of the full golf swing are substantially more demanding than those of putting, involving faster rotations, which very likely increase orientation errors [17]. More recently, Park and Park [25] proposed a personalized kinematic constraint-based drift correction framework for 3D golf swing trajectory monitoring using a wrist-worn IMU. Their method introduced a subject-specific constraint based on the relative wrist displacement between address and impact, estimated from monocular video by compensating depth ambiguity using club length as a geometric constraint. Although this approach reduced trajectory errors to approximately 20 cm over the full swing and 15 cm during the downswing, it also relied on additional video information and on a learning-based framework, which may require task- or subject-specific training and may limit direct generalizability. Other recent IMU-based golf studies further confirm the current interest in inertial sensing for golf analysis, while also showing that full trajectory reconstruction remains relatively underexplored. For example, Vec and Tomažič [26] proposed an algorithm to interpolate corrupted orientation data around the short interval of club–ball impact, but the method was limited to orientation estimation rather than position reconstruction. Similarly, Le et al. [27] presented an on-device smart golf club sensing system using IMU signals and self-supervised learning for club path recognition, but the focus was on motion classification and club path recognition rather than on reconstructing the continuous 3D trajectory of the club.

The present work addresses this challenge through an innovative methodology for putter trajectory reconstruction based on a redundant dual-IMU configuration, in which two sensors are mounted at different locations on the club to improve the robustness of both orientation and position estimation during the putting stroke. The rationale is to exploit the partial independence of the main error sources affecting each sensor, similarly to the strategy proposed in [28], while embedding this redundancy within a new deterministic constrained optimization framework to explicitly model and compensate sensor sources of inaccuracies. In particular, the proposed method introduces a set of constraints on position, velocity, and acceleration variables to reduce drift accumulation, without relying on strong assumptions about a predefined trajectory shape. To express the measurements of both IMUs within a common reference frame and enable their coherent integration, a geometrically realistic model of the putter was developed. The proposed method and the standard reconstruction pipeline were compared and tested on 23 putting strokes using a stereophotogrammetric (SP) system as a reference.

## 2. Materials and Methods

### 2.1. Overview of the Methodological Approach

To analyze drift in IMU-based reconstruction of putter motion, Section 2.2 first introduces the accelerometer and gyroscope measurement models. Section 2.3 then presents the standard pipeline used to estimate position from IMU measurements, together with a simplified mathematical model describing how IMU errors and suboptimal sensor fusion tuning propagate to orientation and position estimates. Section 2.4 introduces an optimization-based pipeline that exploits a redundant two-IMU configuration and task-informed kinematic constraints (CROP) to define and optimize residual error compensation terms and sensor fusion parameter values.

In the following, **bold** symbols are used to denote vector and matrix quantities. One-dimensional time series and rotation matrices are not written in bold.

### 2.2. IMU Measurement Model

**Figure A1:**
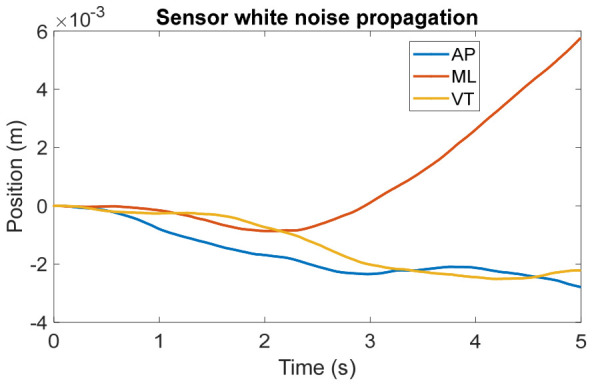
Example of reconstructed position time series obtained from one white noise propagation simulation performed with $\beta =\beta_{grid}=0.001$ rad/s. The AP, ML, and VT components are shown over the five-second simulation interval.

A realistic output of the accelerometer and gyroscope including the main error terms affecting the raw IMU measurements is presented in (1). Ideally, the accelerometer measures the specific force, defined as the difference between the body acceleration, $a_{body}$, and the gravitational acceleration g, expressed in the local coordinate system of the sensor. Similarly, the gyroscope measures the angular velocity of the body around the local sensor axes. In practice, both measurements are affected by deterministic and stochastic error contributions, which are included in the following measurement models:

(1) $$a\left[k\right]=S_{a}\;\left(a_{body}\left[k\right]-g\left[k\right]\right)+b_{a}+\delta b_{a}\left[k\right]+n_{a}\left[k\right]\;\omega \left[k\right]=S_{g}\;\left(\omega_{body}\left[k\right]\right)+b_{g}+\delta b_{g}\left[k\right]+n_{g}\left[k\right]$$

where S accounts for scale factors and non-orthogonality effects, **b** is the constant bias, δb  represents slowly time-varying residual bias components, and n is zero-mean white Gaussian noise. In particular, S and b belong to deterministic errors, which can be effectively compensated through dedicated calibration refinement procedures (e.g., [29,30]). Conversely, δb and n belong to stochastic errors, which cannot be fully removed by standard calibration. To analyze a short-duration trial, the following assumptions are made:

1. For each sensor, S and **b** are considered effectively compensated by the calibration coefficient refinement procedure described in [29].
2. The zero mean white noise term, n, propagates through the reconstruction pipeline because of the integration steps, potentially leading to random walk effects in orientation, velocity, and position. However, its contribution can be neglected if the movement under analysis is limited to a few seconds. Further details and numerical proof are provided in Appendix A.1 (Figure A1 and Table A1).
3. The bias instability δb represents the most critical sensor error component since it models slow and unpredictable fluctuations of the sensor bias arising from the effects of mechanical and electronic components within the sensor chip, which are responsible for run-to-run variations [31,32,33]. However, considering a trial lasting a few seconds, these fluctuations can be reasonably assumed to be constant within a single trial, while still varying across different trials. Under this assumption, the stochastic bias instability can be modeled as an unknown constant residual term for each run, denoted by Δb.

Based on these assumptions, the considered accelerometer and gyroscope output measurement models are described as in (2):

(2) $$a\left[k\right]\approx a_{body}\left[k\right]-g\left[k\right]+{∆b}_{a}\;\omega \left[k\right]\approx \omega_{body}\left[k\right]+{∆b}_{g}$$

### 2.3. Standard IMU-Based Position Estimation Pipeline

**Figure 2:**
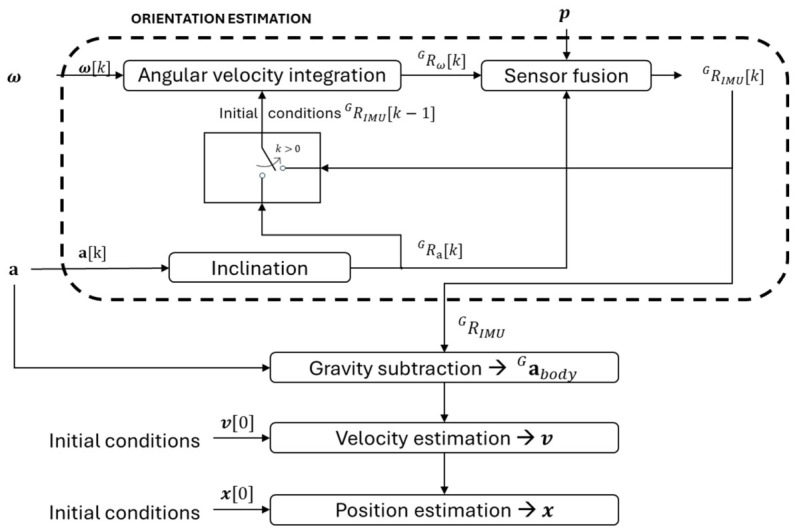
Standard IMU-based reconstruction pipeline used to estimate orientation, velocity, and position from accelerometer and gyroscope measurements.

The standard IMU-based pipeline to estimate orientation, velocity, and position from a and ω time series is presented in Figure 2.

#### 2.3.1. Orientation Estimation and Sensor Measurement Errors Propagation

The absolute orientation of the IMU, RIMUG, is estimated through a sensor fusion algorithm that combines a and ω. In this work, the global reference system G is defined with its vertical axis (z) aligned with the upward gravity direction, and its horizontal x-axis aligned with the principal direction of the movement under analysis.

The orientation estimation process starts from ω, which is integrated over time to provide the relative change in orientation RωGk. As reported in (3), the integration is performed using the previous orientation estimate, RIMUG[k−1], as the initial condition, where Ω(ω[k]) denotes the skew symmetric matrix associated with ω[k]. For k=0, the previous orientation estimate is not available. Hence, RIMUG[k−1] is initialized using the accelerometer-based inclination estimate, RaG[0], typically computed during the static phase preceding the movement. The term Δt represents the sampling interval.

(3) RωGk=expΩωkΔt RIMUG[k−1].

The accelerometer contribution consists of exploiting the gravity direction $g\left[k\right]$ to compute the IMU inclination, RaGk, used to compensate for the drift errors affecting RωGk. Equation (4) models the computation of RaGk:

(4) RaGk=Iak,

where I denotes the specific trigonometric mapping adopted to convert the measured gravity direction g to the corresponding inclination angles. It is worth noting that when not in the static phase, the $a_{body}$ component of the accelerometer output, being included in a, can be considered as a disturbance term, since it is superimposed on g. This means that in the static phase the inclination is very reliable, while in the dynamic phase the accuracy of RaGk is strongly dependent on the magnitude and direction of $a_{body}\left[k\right]$.

The purpose of the sensor fusion process is to combine the complementary information provided by RaGk and RωGk. From a conceptual point of view, this process can be interpreted as a weighted combination of the two contributions, where their relative importance depends on their reliability under the specific experimental conditions [34]. For example, in high dynamic conditions and for low values of ${∆b}_{g}$, the RωGk contribution is more reliable than RaGk. A simplified single-parameter (0≤γ≤1) formulation of the weighing process, originally proposed by Madgwick et al. [35], is presented in (5). Since the direct weighted sum of rotation matrices does not necessarily belong to SO(3), in practical implementations, the fused orientation is constrained to remain a valid rotation, for example, by operating a normalization step, as in Madgwick’s formulation.

(5) RIMUGk=γRaGk+1−γRgGk.

When γ=0, the estimate relies entirely on the gyroscope, whereas when γ=1, it relies entirely on the accelerometer. In the Madgwick implementation, however, the balance between these two information sources is not implemented explicitly through a direct weighting factor γ, but indirectly through the parameter β, which regulates the contribution of the gradient-descent correction term within the gyroscope-based update equation. Larger values of β increase the influence of accelerometer-based correction, while smaller values favor gyroscope propagation. An a priori proper selection of this sensor fusion parameter value, in general, is not trivial as it depends on different factors, as highlighted in [17,28,36,37], such as specific IMU hardware model, magnitude of the movement dynamics, and specific sensor fusion algorithm implementation.

To illustrate the error propagation within this orientation estimation pipeline, a simplified one-dimensional case is considered. In the following equations, the measurement error terms associated with the accelerometer and the gyroscope are explicitly propagated to show how they affect the estimated orientation. The integration error affecting RωGk can be modelled as follows:

(6) $$e_{\omega }\left[k\right]=\sum_{j=1}^{k}{∆b}_{g}\left[j\right]\Delta t+\;\theta_{{i.c.}_{e}}=\Delta b_{g}\left(k\Delta t\right)+\;e_{SF}\left[k-1\right],$$

where $\Delta b_{g}\Delta t$ represents the linear angular drift due to the integration of the gyroscope bias constant residual term ${∆b}_{g}$ over a period Δt. The term $e_{SF}[k-1]$ accounts for the error already present in the previous orientation estimate, which is used as the initial condition for the gyroscope-based integration at sample k. To provide an intuitive description of accelerometer-based error propagation in the orientation estimation process, an illustrative simplified formulation is considered in the tilt plane, namely along the accelerometer axis sensing the gravity projection. Under a small angle approximation, the mono-dimensional inclination angular errors affecting RaGk can be expressed as

(7) $$e_{a}\left[k\right]=\frac{{∆b}_{a}}{g}.$$

This term represents the contribution of the residual constant accelerometer bias, ${∆b}_{a}$. It should be noted that this is a simplified interpretation aimed at clarifying the propagation mechanism rather than providing a fully rigorous description. In practice, as already mentioned, a major source of accelerometer-related orientation error is also the $a_{body}$ component, which, outside static conditions, is superimposed on g and can significantly affect the inclination estimate. However, the present analysis focuses specifically on sensor measurement errors. For this reason, the contribution of $a_{body}$ is not explicitly propagated here, consistently with common sensor fusion formulations, such as [35], in which it is not explicitly modeled. For this reason, $e_{a}\left[k\right]$ can be considered constant over time.

For the purpose of modelling the orientation error affecting RIMUG after the sensor fusion process, $e_{SF}\left[k\right]$, a simplified equivalent error model is adopted. Errors can enter the sensor fusion process through $e_{a}$ and $e_{\omega }$. Since the exact analytical propagation of these errors through the orientation estimation is complicated by the fact that the sensor fusion process is recursive and involves nonlinear operations, an equivalent additive formulation is adopted:

(8) $$e_{SF}\left[k\right]\sim c_{a}\left[k\right]e_{a}\left[k\right]+c_{\omega }\left[k\right]e_{\omega }\left[k\right],$$

where $c_{a}\left[k\right]$ and $c_{\omega }\left[k\right]$ are equivalent weighting factors that summarize the relative influence of $e_{a}$ and $e_{\omega }$. If the two error contributions are assumed to be constant over the analyzed time window, the sample dependence can be omitted and the simplified sensor fusion error model becomes

(9) $$e_{SF}\left[k\right]\sim c_{a}e_{a}+c_{\omega }e_{\omega }=c_{a}\frac{{∆b}_{a}}{g}+c_{\omega }\Delta b_{g}\Delta t.$$

This approximation is not intended to describe the sample-by-sample dynamics of the filter, but to provide a compact expression for analyzing how residual orientation errors propagate through the subsequent velocity and position reconstruction steps.

#### 2.3.2. Position Estimation and Sensor Measurement Errors Propagation

The estimated orientation RIMUGk is then used to subtract the gravity vector from the accelerometer output expressed in the global reference system to isolate abodyG, as follows:

(10) abodykG=RIMUGtiak+gG.

Then, abodykG is first integrated to obtain the velocity $v\left[k\right]$, given the integration initial conditions, $v\left[0\right]$.

(11) vk=∑j=1kabodyGjΔt+v0.

Finally, $v\left[k\right]$ is integrated to obtain the position $x\left[k\right]$, given the integration initial conditions, $x\left[0\right]$.

(12) $$x\left[k\right]=\sum_{j=1}^{k}v\left[j\right]\Delta t+x\left[0\right].$$

Similarly to what was done for orientation estimation, a simplified error propagation analysis is introduced here to highlight how the main measurement and initialization errors affect position reconstruction. Errors affecting RIMUGk are propagated throughout Equations (10)–(12).

The approximate error affecting the body acceleration, $e_{a_{body}}$, can be assumed in the worst case as

(13) $$e_{a_{body}}\left[k\right]\sim \;e_{SF}\left[k\right]\;g.$$

Errors affecting velocity, $v_{err}$, can be formulated as follows:

(14) $$v_{e}\left[k\right]=\sum_{j=1}^{k}e_{a_{body}}\left[j\right]\Delta t+\;v_{0err}=gc_{\omega }\Delta b_{g}{\left(k\Delta t\right)}^{2}+c_{a}{∆b}_{a}\left(k\Delta t\right)+\;e_{v_{0}}.$$

The term $e_{v_{0}}$ corresponds to the error in estimating the initial velocity conditions. Errors affecting position, $x_{e}$, can be formulated as follows:

(15) $$x_{e}\left[k\right]=\sum_{j=1}^{k}v_{e}\left[j\right]\Delta t+x_{0e}=gc_{\omega }\Delta b_{g}{\left(k\Delta t\right)}^{3}+c_{a}{∆b}_{a}{\left(k\Delta t\right)}^{2}+e_{v_{0}}\left(k\Delta t\right)+e_{x_{0}}.$$

The term $e_{x_{0}}$ corresponds to the error in estimating the initial position conditions. From (15), it can be seen that the position error is affected by three main contributions: the residual gyroscope bias $\Delta b_{g}$, which induces a cubic drift amplified by g and modulated by the sensor fusion parameter value, the residual accelerometer bias $\Delta b_{a}$, which induces a quadratic drift, and errors in the initial conditions $e_{v_{0}}$, which propagate linearly over time. This means that even over a short time interval, small residual errors in gyroscope bias, accelerometer bias, and initial conditions can be strongly amplified, leading to unacceptable position reconstruction errors. Therefore, mitigating these residual error sources is essential to improve the robustness of IMU-based trajectory reconstruction.

### 2.4. Constrained Redundant Optimization-Based Position Estimation Pipeline (CROP)

**Figure 3:**
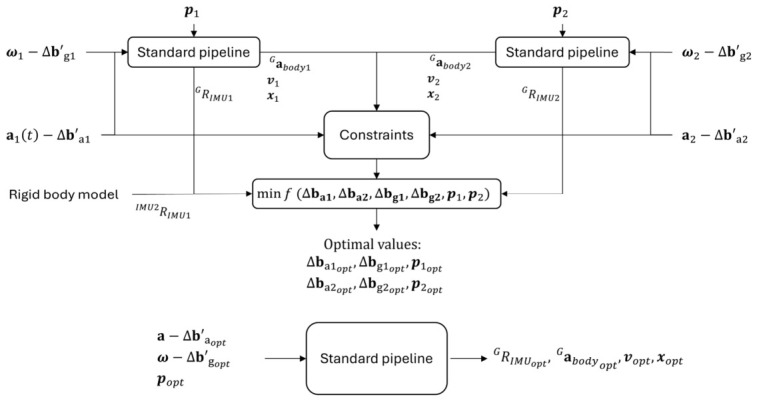
The CROP pipeline which optimizes the residual bias compensation terms $\Delta b_{a}^{\prime }$ and $\Delta b_{g}^{\prime }$, together with the sensor fusion parameters p for each IMU. The optimized parameters are then used in the standard pipeline to reconstruct orientation, velocity, and position.

To address these limitations, this section introduces CROP, an optimization-based pipeline with task-informed kinematic constraints designed to estimate and mitigate the residual error sources that most affect position reconstruction. The proposed approach, presented in Figure 3, exploits the redundancy provided by a dual-IMU configuration mounted on the same rigid body and optimizes, for each IMU, residual sensor error mitigation terms, namely $\Delta {b\prime }_{a}$ and $\Delta {b\prime }_{g}$, together with the sensor fusion parameters p.

Let ${IMU}_{1}$ and ${IMU}_{2}$ denote the two units mounted on the same rigid body. As shown in Figure 3, the standard reconstruction pipeline is retained as the core processing module, but it is embedded within a constrained optimization framework. For each candidate set of optimization variables, the measurements of each IMU are first corrected. Then, the orientation of each IMU is estimated independently. Finally, the resulting orientation, acceleration, velocity, and trajectory variables are obtained through the standard pipeline to evaluate both the objective function and the nonlinear constraints.

In further detail, for each IMU, two residual bias terms, $\Delta {b\prime }_{a}$ and $\Delta {b\prime }_{g}$, are introduced to subtract the residual bias from the accelerometer and gyroscope measurements. In addition, the sensor fusion parameter p is included among the optimization variables, to allow for proper tuning of the orientation filter parameter. Accordingly, the complete set of optimization variables is defined as $\Delta {b\prime }_{a1},\Delta {b\prime }_{a2},\Delta {b\prime }_{g1},\Delta {b\prime }_{g2},p_{1},p_{2}$. For a given value of these variables, the corrected accelerometer and gyroscope signals are processed by the standard pipeline, producing the orientation estimates RIMU1Gk and RIMU2Gk.

The objective function f is defined according to the rigid body constraint method introduced in [28,36]. The rationale is to exploit the partial independence of the main error sources affecting two IMUs mounted at different locations on the putter, similarly to the strategy proposed in [28]. Since both IMUs are mounted on the same rigid body, their relative orientation should remain constant over time. Therefore, any variation in the relative rotation between the two units can be interpreted as an inconsistency caused by residual sensor errors and non-optimal sensor fusion parameters. To compute their relative rotation error, the orientations of the two IMUs are expressed in the same reference frame using the time invariant RIMU1IMU2, provided by the geometric modelling of the rigid body. At each time instant $t_{i}$, the residual relative rotation is computed as

(16) Rek=RIMU2GkRIMU1IMU2TRIMU1Gk. 

In the ideal case, $R_{e}\left[k\right]$ should be equal to the identity matrix. The corresponding angular error $\varphi_{e}\left[k\right]$ is obtained by converting $R_{e}\left[k\right]$ into its angular scalar quantity following the axis-angle representation. The objective function f is then defined as the root mean square (rms) of $\varphi_{e}\left[k\right]$ as follows:

(17) $$f\left(\Delta {b\prime }_{a1},\Delta {b\prime }_{a2},\Delta {b\prime }_{g1},\Delta {b\prime }_{g2},p_{1},p_{2}\right)=rms\;\left(\varphi_{e}\left[k\right]\right).\;$$

The optimization aims to identify the optimal set for each IMU, i.e., $\Delta {b\prime }_{a1_{opt}},\Delta {b\prime }_{g1_{opt}},p_{1_{opt}}$ and $\Delta {b\prime }_{a2_{opt}},\Delta {b\prime }_{g2_{opt}},p_{2_{opt}}$, that minimizes f.

To further improve robustness of the minimization process, a set of nonlinear task-specific constraints *C* can be introduced to account for the quantities involved in the reconstruction process, from the corrected inertial signals to the derived acceleration, velocity, and position variables. In this way, optimization is not driven solely by the rigid body orientation error, but it is reinforced by additional kinematic conditions that help identify the most appropriate set of residual bias terms and sensor fusion parameters. Moreover, the constraints also reduce the admissible search space, improving the robustness of the optimization and reducing the computational burden. Constraints defined on acceleration variables act directly on the corrected accelerometer signals and therefore limit the admissible values of the residual accelerometer bias compensation terms $\Delta {b\prime }_{a}$. Constraints defined on reconstructed velocity and position can impose task-specific biomechanical or geometrical consistency conditions, thus preventing unrealistic trajectories. These constraints restrict the admissible combinations of $\Delta {b\prime }_{a}$, $\Delta {b\prime }_{g}$, and p, avoiding parameter configurations that reduce orientation inconsistencies while producing implausible kinematic reconstructions.

After the minimization, the optimal set of parameters is used in the final execution of the standard reconstruction pipeline, yielding the optimized trajectory estimate.

The proposed method has been made publicly available through the following open-source repository: https://github.com/H-MOVE-LAB/golf_imu (accessed on 18 May 2026).

### 2.5. Club Modelling and Experimental Session

**Figure 4:**
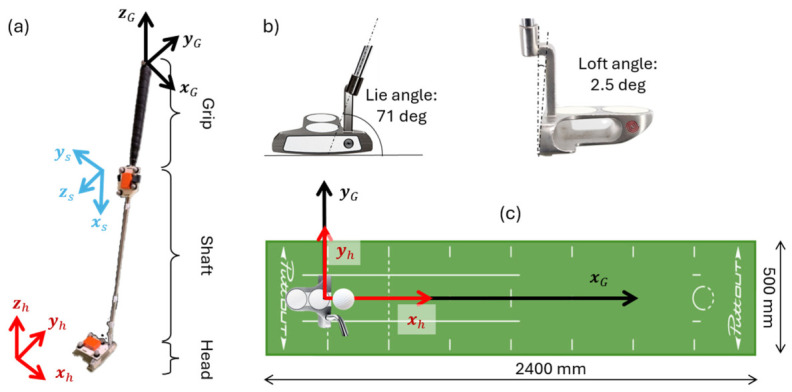
(**a**) The putter instrumented with the shaft and head IMUs mounted on 3D-printed support plates carrying retroreflective markers; (**b**) the lie and loft angles; (**c**) the putting setup on the practice mat and the definition of the global and putter head reference frames.

The putter is a rigid body composed of three main parts: the grip, namely the portion held by the player, the shaft, which connects the grip to the clubhead, and the head, which is the part that strikes the ball (Figure 4a). Although this structure may appear simple at first glance, the geometry of the putter is carefully designed to control how the club interacts with both the ball and the turf. Two angular features are particularly relevant in this respect: lie and loft (Figure 4b). The lie angle determines how the shaft is inclined relative to the ground, and consequently how the clubhead sits at address and moves through the stroke. The loft angle defines the slight inclination of the face with respect to the vertical, which helps the ball leave the grass surface cleanly and start rolling smoothly. For these reasons, the putter cannot be modelled as a simple straight rod. In the present study, the putter Odyssey White Steel 2-Ball Blade (Odyssey Golf, Carlsbad, CA, USA) was adopted, and its geometry was measured accurately using calipers and goniometers to define the rigid body (putter) model.

For the data acquisitions, an amateur golfer (handicap = 17.3, 27-year-old male) was asked to perform 23 strokes using the putter Odyssey White Steel 2-Ball Blade on the PuttOUT practice mat (Figure 4c). One IMU (Xsens–MTw, Xsens Technologies, Enschede, The Netherlands) was mounted on the shaft (${IMU}_{1}$≡${IMU}_{s}$), just below the grip, and one on the club head (${IMU}_{2}$≡${IMU}_{h}$) (Figure 4a). Each IMU measured 47 × 30 × 13 mm, weighed 16 g, and included a 3D accelerometer, ±160 m/s^2^, and a 3D gyroscope, ±2000 deg/s. The measurements of both IMUs were expressed within a common reference frame through the putter model, consisting of the time-invariant RIMU1IMU2. A five-minute warm up period was ensured before data acquisition to minimize temperature-related variability in the IMU outputs. IMU data of all the recorded trials have been made publicly available at https://github.com/H-MOVE-LAB/golf_imu (accessed on 18 May 2026).

An SP system (Vicon–Vero, Vicon, Yarnton, UK) was used as a reference, consisting of 12 infrared cameras and 8 retroreflective passive markers. The IMUs were mounted using 3D-printed plastic support plates. Each support hosted one IMU and four retroreflective passive markers, with a diameter of 14 mm, used to provide reference orientation and trajectory. Each support was specifically designed with four slots to ensure accurate alignment between the IMU and marker local coordinate systems, enabling direct comparison between the two measurement systems. The plate on the shaft was secured with Velcro straps and elastic bands, while the plate on the putter head was attached using double-sided adhesive tape (Figure 4a). IMU and SP systems collected data at 100 Hz and were synchronized by means of a trigger through two BNC-RCA cables to ensure time alignment accuracy within 1 sample (±10 ms). The total mass of each complete mounting assembly, including the IMU, the 3D-printed support plate, and the four retroreflective markers, was about 50 g.

### 2.6. Data Analysis for Golf Putting

Data analysis was performed entirely in MATLAB R2025b (The MathWorks Inc., Natick, MA, USA). Before trajectory reconstruction, the calibration refinement coefficients estimated according to the procedure described in [29] were applied to the raw accelerometer and gyroscope measurements. These coefficients were used to compensate for the deterministic error terms introduced in (1), namely scale factors and non-orthogonality effects, represented by S, and constant bias, represented by b.

For both implemented methods (standard pipeline and CROP), orientation, velocity, and position were estimated in the chosen global reference frame *G* (Figure 4a). The latter was defined with its vertical axis (z) aligned with the upward gravity direction, and its horizontal x-axis aligned with the direction normal to the club face during the static phase preceding the movement projected onto the horizontal plane. The initial velocity was set to zero at the beginning of the reconstruction, consistently with the manually segmented static interval preceding the stroke to reduce the effect of initial velocity errors on the subsequent trajectory reconstruction, whose influence was highlighted in (15).

For each trial, head-mounted IMU data were first processed using the standard reconstruction pipeline. The sensor fusion algorithm proposed by Madgwick et al. [35] was selected because it relies on a single tuning parameter, β, which regulates the relative contribution of the accelerometer-based correction with respect to gyroscope integration. In particular, β=0 rad/s corresponds to a purely gyroscope-driven orientation update, with no accelerometer-based correction after initialization. The first two values, β=0 rad/s and β=0.2 rad/s, were selected to evaluate the two extremes of a reasonable tuning interval. The third β = $\beta_{grid}$ value was identified through a grid search procedure as the value that minimized the orientation error across all recorded trials. This single *β* value, corresponding to the best-performing global configuration across the dataset, was then applied to all trials to reflect a realistic deployment scenario without trial-specific tuning. This latter condition was included in evaluating the standard pipeline under its best achievable tuning, thus providing a more favorable benchmark for comparison with CROP.

The same trials were then processed using CROP. The optimization problem was solved using a sequential quadratic programming algorithm. To mitigate the risk of convergence to local minima, a multi-start approach with seven runs was adopted, representing a compromise between solution-space exploration and computational cost. A set of nonlinear constraints *C* specific to the putting stroke task was incorporated into the constrained optimization problem by means of Lagrange multipliers. For each IMU, the following constraints were imposed during the static phases before and after the stroke (referred to as $t_{static}$):

- $C_{1}$: the magnitude of the average accelerometer signal, $\left\|\bar{a}(t_{static})\right\|,$ is constrained to lie within $g\;\pm 0.01\;m/s^{2}$;
- $C_{2}$: the average acceleration, ${\bar{a}}_{body}(t_{static})$, is equal to ${0\;m/s}^{2}$ for each axis;
- $C_{3}$: the average velocity, $\bar{v}(t_{static})$, is equal to 0 m/s for each axis;
- $C_{4}$: the maximum of the absolute value of the velocity, $\max\left|v(t_{static})\right|$, is constrained to lie within 0 m/s and 0.005 m/s for each axis.

During the dynamic phase ($t_{dyn}$), the following constraint was imposed:

- $C_{5}$: the position of the putter head along the vertical axis, $p_{1,VT}(t_{dyn})$, is constrained to lie within 0 m and 0.1 m.

The numerical thresholds associated with the optimization constraints were selected a priori from expected sensor noise levels and biomechanical considerations of the putting movement. The vertical displacement bound (0–0.1 m) was chosen to encompass the expected range of putter-head motion during putting while excluding clearly non-physical solutions caused by orientation or integration drift.

The optimization variables included $\Delta {b\prime }_{a}$, $\Delta {b\prime }_{g}$, and β′, for each IMU. To improve computational efficiency and to prevent the optimizer from exploring physically unreasonable solutions, bounded search intervals were imposed on all optimization variables. For each IMU, $\Delta {b\prime }_{a}$ was constrained between $\left[-0.2,\;0.2\right]\;{m/s}^{2}$ along each axis, $\Delta {b\prime }_{g}$ was constrained between $\left[-0.1,\;0.1\right]$ deg/s along each axis, and β′ was constrained between [0, 0.2] rad/s. The optimized residual bias terms and sensor fusion parameters were used to run the final reconstruction pipeline. Finally, the optimized values of $\Delta {b\prime }_{a}$, $\Delta {b\prime }_{g}$, β′ were graphically inspected to verify that they did not systematically converge to the imposed bounds and that they varied across trials and IMUs, consistently with the expected run to run variability.

Computational time was measured for each trial during the execution of CROP on a laptop equipped with an Intel Core Ultra 9 185H processor (Intel Corporation, Santa Clara, CA, USA), 32 GB RAM, 2 × 16 GB LPDDR5x, 6400 MT/s, and Windows 11 Home (Microsoft Corporation, Redmond, WA, USA).

### 2.7. Method Performance Evaluation

Orientation errors were computed by comparing both IMU-based methods (standard pipeline and CROP) and reference SP orientation time series. The SP marker trajectories were first labeled and gap-filled in Nexus (Vicon–Vero, Vicon, Yarnton, UK). After gap filling, marker trajectories were low-pass-filtered using a zero-phase sixth-order Butterworth filter with a cut-off frequency of 6 Hz, as recommended for movement analysis applications [38]. For each support plate, the reference position was defined as the centroid of the four corresponding retroreflective markers. The reference orientation was obtained by defining the local coordinate system of the marker cluster and computing its orientation with respect to the SP global coordinate system using an SVD-based rigid-body fitting procedure [39]. Based on the size of the marker cluster and on the expected marker position error of the SP system, the reference orientation error was assumed to be approximately 0.5 deg [28,40]. For each IMU-based method, at each sample, the relative rotation matrix between the two estimates was computed and converted into the corresponding scalar angular error using the axis angle representation, $e_{or}$. The root mean square (RMS) value of $e_{or}$ was then used as an orientation error metric. Position errors were computed by comparing each IMU-based reconstructed trajectory with the reference trajectory obtained from SP. The instantaneous position error was calculated as the point-by-point Euclidean distance between the two trajectories ($e_{x,norm}$), together with the axis-wise absolute errors along the three global axes, namely antero-posterior, medio-lateral, and vertical ($e_{x,AP},\;e_{x,ML},\;e_{x,VT}$). RMS values were computed for $e_{x,AP},\;e_{x,ML},\;e_{x,VT}$, and $e_{x,norm}$. Additionally, the maximum value of $e_{x,norm}$ was computed. Velocity errors were computed analogously, by comparing the IMU-based velocity estimate with the reference velocity obtained by computing the time derivative of the SP centroid trajectory. Thus, four velocity errors were obtained, $e_{v,norm}$, $e_{v,AP},\;e_{v,ML},\;e_{v,VT}$, and their RMS values were computed, as well as the maximum value of $e_{v,norm}$.

## 3. Results

The orientation parameter value that provided the best performance for the standard pipeline, as identified through grid search, was $\beta_{grid}=0.001$ rad/s.

Median values, together with the 25th and 75th percentiles, of the orientation, velocity, and position errors obtained from the standard pipeline, applied with the three selected β values, and from CROP are reported in Table 1. Errors for each trial are reported for standard pipeline with $\beta_{grid}$ and for CROP in Table A2 and Table A3 in Appendix A.2, respectively.

**Figure 5:**
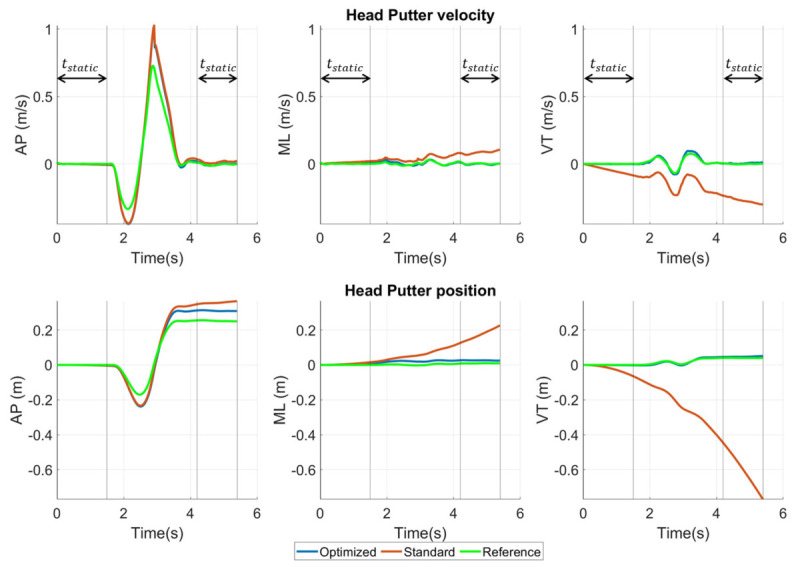
Illustrative velocity and position time series of the putter head obtained with the standard pipeline using $\beta_{grid}$, with CROP, and from the reference SP system. The upper panels show the anteroposterior (AP), mediolateral (ML), and vertical (VT) velocity components, while the lower panels show the corresponding position components. Vertical gray thick lines indicate the manually segmented static intervals before and after the dynamic swing phase ($t_{static}$).

An example of velocity and position time series obtained using the standard pipeline with $\beta_{grid}$, and CROP is shown against the reference in Figure 5 to provide a graphical comparison.

**Figure 6:**
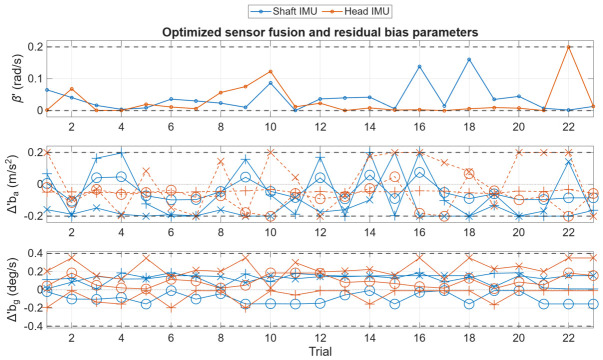
Optimized residual bias compensation terms and sensor fusion parameters obtained with CROP for the shaft and head IMUs across the 23 trials. The panels show, from top to bottom, the optimized β′, $\Delta {b\prime }_{a}$, and $\Delta {b\prime }_{g}$ values. Dashed horizontal lines indicate the imposed search bounds for each parameter set. Markers “+”, “x”, and “o” refer to x, y, and z axes, respectively.

Figure 6 shows the optimized values of $\Delta {b\prime }_{a}$, $\Delta {b\prime }_{g}$, β′ for each IMU across trials. Dashed horizontal lines represent the search limit for each set of parameters.

The average computational cost to process a single trial through CROP was 25.3 ± 11.8 s.

## 4. Discussion

This study proposed the CROP pipeline, aimed at improving IMU-based position reconstruction of a rigid body through a redundant (dual-IMU) and constrained (task-specific) optimization approach. This pipeline minimized a cost function based on the orientation difference between the two IMUs to optimally set the most relevant parameters of the standard IMU-based pipeline for position reconstruction (i.e., orientation parameter and residual inertial biases). This proposed pipeline was applied to golf putting for putter head trajectory reconstruction and compared to the standard IMU-based pipeline. To this end, the study analyzed how errors propagate in the standard IMU-based pipeline for putter position reconstruction and identified the main sources of trajectory errors.

As expected [16,17], orientation errors obtained with the standard pipeline showed a strong dependence on the selected value of β. The grid search identified a $\beta_{grid}$ value very close to 0 rad/s (i.e., $\beta_{grid}$ = 0.001 rad/s), suggesting that, over the short duration of the analyzed movement, gyroscope integration can be sufficiently reliable for orientation estimation. Consistently, the median RMS orientation error obtained with β=[0,0.001] rad/s amounted to 1.5 deg. Conversely, when β=0.2 rad/s, the median orientation errors increased to 5.2 deg. This result indicates that assigning a higher weight to the accelerometer-based correction was not suitable for the analyzed movement, likely because this inclination estimate was affected by the dynamic body acceleration of the putter. Orientation errors obtained with CROP were similar to those obtained with $\beta_{grid}$, as orientation difference below 0.5 deg cannot be considered relevant [28]. This similarity was expected, since both configurations were designed to reduce orientation inconsistency.

CROP provided a clear improvement in both velocity and position reconstruction. Considering the RMS of the velocity norm, CROP yielded a median error of 9.9 cm/s, compared with 17.1 cm/s, 16.5 cm/s, and 74.3 cm/s for the standard pipeline with β=0 rad/s, $\beta =\;\beta_{grid}$, and β=0.2 rad/s, respectively. Thus, even when the standard pipeline was evaluated using the best grid-searched value of β, CROP reduced the median velocity norm error by approximately 40%.

A similar improvement was observed for position reconstruction. The median RMS error of the position norm decreased from 23.2 cm with $\beta_{grid}$ to 8.5 cm with CROP, corresponding to an approximately 63% reduction. The maximum position norm error was also reduced, from 51.2 cm to 14.8 cm, corresponding to an approximately 70% reduction.

The improvement was also consistent across individual trials (Appendix A.2). Compared with the standard pipeline using $\beta =\beta_{grid}$, CROP reduced the RMS error of the position norm in 22 out of 23 trials. The largest trial RMS error decreased from 0.465 m with the standard pipeline to 0.190 m with CROP. The same trend was observed for the maximum position norm error, which was also lower with CROP in 22 out of 23 trials.

The axis-wise results provide additional insight into the effect of the optimization (Appendix A.2). In the standard pipeline, the distribution of errors across the AP, ML, and VT components changed depending on the selected value of β, confirming that sensor fusion tuning affects not only the overall error magnitude but also the direction along which drift mainly develops. After CROP, the error distribution showed a marked reduction along the VT component. This result is particularly relevant because the vertical motion of the putter head is more strongly constrained by the task geometry and was explicitly controlled in the optimization. Conversely, the AP component is less suitable for strict position constraints, since it represents the main direction of motion and is inherently more variable across trials.

These results show that minimizing the overall orientation error alone was not sufficient to guarantee accurate trajectory reconstruction. The standard pipeline tuned through grid-search achieved the same orientation errors as CROP, but its position and velocity errors remained substantially larger than those obtained with CROP. A qualitative example of this behavior is shown for one trial in Figure 5. The estimates obtained with the standard pipeline progressively diverged from the reference velocity and position trajectories, while the optimized pipeline provided a closer agreement throughout the analyzed movement. This supports the rationale of the proposed approach, in which the optimization was not driven only by orientation consistency, but was reinforced by reasonable constraints on the reconstructed kinematic variables.

The optimized parameters varied across trials and between the two IMUs, as highlighted in Figure 6, consistently with the adopted measurement model. In particular, the optimized values were distributed within the imposed search intervals and did not show a systematic accumulation at the lower or upper bounds. In this framework, the residual accelerometer and gyroscope bias terms were not assumed to be fixed deterministic quantities, but rather residual components that could vary across trials because of run-to-run sensor variability and trial-specific acquisition conditions. As a result, the optimal values of $\Delta b_{a}^{\prime }$ and $\Delta b_{g}^{\prime }$ were expected to change from one recording to another. The variability observed in the optimized $\beta^{\prime }$ values is also coherent with this interpretation, as its optimal value depended on the relative magnitude of the residual errors affecting the two information sources. Overall, these results indicate that the optimization adapted the residual bias compensation and sensor fusion tuning to the specific error conditions of each trial, supporting the need for an adaptive redundant framework rather than a fixed calibration or a single global sensor fusion parameter.

Overall, these findings indicate that the proposed optimization effectively reduced the drift affecting the standard reconstruction pipeline, although accurate and fully reliable putter trajectory reconstruction under field conditions remains an open challenge. A universal threshold defining acceptable position reconstruction accuracy for this application cannot be clearly established, since it depends on the intended use and on the specific outcome of interest. Moreover, commercial putting analysis systems generally report specific stroke parameters, sensor resolution, or impact-zone measurements rather than full-trajectory RMS errors directly comparable with the metric used in the present study. Therefore, the achieved median RMS value of 8.5 cm should be interpreted as still substantial for applications requiring a detailed quantitative analysis of the complete putter head trajectory. King et al. reported millimeter-level errors, but their validation was performed under highly controlled conditions using a single degree of freedom pendulum robot. Similarly, Burchfield and Venkatesan [22] obtained position errors lower than 1 cm by constraining the club trajectory to a prescribed parabolic path, an assumption that is not necessarily valid for real putting movements. On the other hand, Kim et al. [24], using a less constrained setting for golf swing reconstruction, reported trajectory errors in the order of 17 cm despite combining an IMU with kinematic constraints and a deep-learning based model. In this context, CROP represents an intermediate step: it improves over the unconstrained standard IMU pipeline without imposing a fixed athlete-independent trajectory, but further work is required to reach field-ready accuracy. At the same time, its modular structure may represent a relevant advantage, since additional constraints could be incorporated. The same framework could, in principle, be adapted to other movement analysis applications in which redundant IMU configurations and task specific kinematic constraints can be exploited to reduce drift. However, such an extension would require application specific validation. In particular, the selected IMUs should have input ranges and bandwidths adequate for the expected movement dynamics, to avoid saturation during high-dynamic phases such as the downswing phase of a full golf swing [25] or high-speed racket sports [41]. In addition, the sensorized setup should be sufficiently compact and lightweight so as not to interfere with the natural execution of the gesture, whereas the task specific constraints should remain informative but not overly restrictive, so that they can account for the natural variability of the movement.

Some limitations should be acknowledged. First, the proposed approach relied on the assumption that the two IMUs are mounted on the same rigid body. This assumption is reasonable for putting, where club deformation is limited, but it may not hold during highly dynamic movements such as the downswing of a full golf swing, where shaft deformation can become relevant. In addition, the identification of static or zero velocity phases was more straightforward in putting than in faster and more continuous golf gestures.

A second limitation concerns the initialization of velocity. In this study, the initial velocity was assumed to be zero, consistent with the static interval preceding the stroke. As shown in Equations (14) and (15), errors in the initial velocity propagate over time and can therefore affect the reconstructed position. Incorrect identification of the zero-velocity interval may consequently introduce reconstruction errors. Moreover, since constraints $C_{1}$–$C_{4}$ relied on average quantities computed during static intervals, incorrect static segmentation may violate the constraint assumptions and lead to inaccurate optimized parameters. To limit this issue, static intervals were manually segmented in the present work. Future implementations should investigate automatic and robust procedures for detecting reliable zero-velocity intervals.

Another limitation concerns the additional mass introduced by the two sensorized mounting assemblies attached to the shaft and club head. Although the golfer did not report perceiving a relevant alteration of the stroke during the acquisitions, the possible influence of the added mass on natural putting execution was not quantitatively assessed. Therefore, the present setup should be interpreted as a laboratory proof of concept for technical validation of the reconstruction pipeline. Future implementations should minimize this effect by using lighter hardware or by integrating the sensors directly into the club structure.

A further limitation is related to the sensor full-scale setting. Signal saturation occurred during the most demanding ball-striking phases, as reported in previous IMU-based golf swing studies [25]. Nevertheless, the effect on the results is expected to be minimal because saturation occurred only over a very brief period, corresponding to approximately one sample.

The method is computationally more demanding than the standard pipeline, since it requires solving a constrained optimization problem for each acquisition. In the present implementation, the average computational time required to process one trial with CROP was 25.3 ± 11.8 s. Accordingly, the method is not suitable for real-time feedback, but it remains compatible with offline analysis or with delayed feedback provided a few tens of seconds after each stroke or at the end of a training session. Future work should improve computational efficiency by optimizing the code implementation, exploring parallelization, and investigating more efficient solver configurations.

Finally, the experimental validation was performed on a single golfer and using one IMU hardware model. Therefore, the present results should be interpreted as a proof-of-concept technical validation of the proposed reconstruction framework, rather than as a complete assessment of its generalizability across golfers, skill levels, putting styles, putters, and sensor models. Differences in movement patterns and equipment characteristics may affect both the error propagation mechanisms and the effectiveness of the optimized parameters. Since residual sensor instability and bias variability are known to depend on the specific hardware [17,37], the performance of CROP may vary with different IMU models. Future work should include cohorts of golfers with different putting techniques, different putters, and different IMU hardware to assess the generalizability of the proposed approach.

## 5. Conclusions

This work proposed CROP, a task-informed constrained optimization pipeline that exploits two IMUs mounted on the same rigid body, IMU redundancy, and kinematic constraints to estimate trial-dependent residual sensor error terms and sensor fusion parameters, rather than relying on fixed calibration and predefined filter tuning. Compared with the best-tuned standard pipeline, CROP reduced the median RMS error of the velocity norm from 16.5 cm/s to 9.9 cm/s, corresponding to an approximately 40% reduction. For position reconstruction, the median RMS error of the norm decreased from 23.2 cm to 8.5 cm, corresponding to an approximately 63% reduction. These results indicate that the proposed optimization effectively reduces the drift affecting the standard pipeline and improves the reconstruction of the kinematic quantities most affected by error accumulation. Despite the increased computational cost with respect to a standard pipeline, CROP supported the feasibility of using IMUs as low-cost tools for golf putting analysis by improving the reliability of IMU-based putter trajectory reconstruction.

Although the achieved accuracy represents a clear improvement over the standard pipeline, further methodological improvement and validation under broader experimental conditions are still required before the approach can be considered fully reliable for field-based putter trajectory reconstruction. Nevertheless, the proposed CROP framework may represent a general approach for mitigating IMU-related errors in rigid-body position reconstruction, as its modular structure allows the integration of additional task-specific constraints depending on the characteristics and biomechanical requirements of the analyzed movement.

For reproducibility purposes, the MATLAB (R2025b) implementation of the proposed framework, together with the experimental dataset used in this study, has been made publicly available as open source.

## Figures and Tables

**Table 1:** Orientation, velocity, and position reconstruction errors obtained with the standard pipeline using the three selected values of β, and with CROP. Values are reported as median [25th, 75th percentiles] across the 23 trials. Velocity and position errors are reported along the anteroposterior, AP, mediolateral, ML, and vertical, VT, axes, and as norm-based errors.

		Median Error [25th, 75th]			
		Standard Pipeline			CROP
		β=0 rad/s	β=0.2 rad/s	$\beta =\beta_{grid}$ rad/s	
Orientation (deg)	$\mathrm{RMS}\;(e_{or}$)	1.5 [1.2, 2.5]	5.2 [4.5, 5.7]	1.5 [1.2, 2.5]	1.7 [1.2, 2.7]
Velocity (cm/s)	$\mathrm{RMS}\;(e_{v,AP}$)	9.3 [7.3, 10.6]	73.4 [45.4, 82.7]	9.4 [7.4, 10.8]	9.8 [8.5, 11.3]
	$\mathrm{RMS}\;(e_{v,ML}$)	3.7 [2.5, 5.7]	3.6 [2.0, 4.3]	2.9 [2.3, 4.9]	1.0 [0.8, 1.7]
	$\mathrm{RMS}\;(e_{v,VT}$)	13.3 [11.0, 15.4]	12.2 [10.7, 14.8]	13.3 [11.0, 15.4]	1.2 [1.0, 1.9]
	$\mathrm{RMS}\;(e_{v,norm}$)	17.1 [15.7, 18.8]	74.3 [47.3, 83.6]	16.5 [15.6, 18.6]	9.9 [8.6, 12.0]
	$\mathrm{MAX}\;(e_{v,norm}$)	34.0 [32.1, 36.0]	121.9 [86.3, 137.2]	33.4 [32.2, 36.3]	33.4 [32.2, 40.3]
Position (cm)	$\mathrm{RMS}\;(e_{x,\;\;AP}$)	6.1 [ 4.4, 7.6]	68.4 [ 44.8, 88.4]	6.7 [5.3, 8.2]	8.4 [6.5, 12.4]
	$\mathrm{RMS}\;(e_{x,ML}$)	4.3 [ 2.2, 7.2]	3.1 [1.1, 4.3]	3.2 [2.4, 6.2]	1.0 [0.6, 1.6]
	$\mathrm{RMS}\;(e_{x,VT}$)	21.0 [15.1, 29.0]	17.1 [10.4, 23.8]	21.0 [15.1, 29.0]	0.7 [0.5, 1.1]
	$\mathrm{RMS}\;(e_{x,norm}$)	23.1 [17.3, 30.3]	69.9 [ 51.6, 91.6]	23.2 [17.4, 30.2]	8.5 [6.6, 12.5]
	$\mathrm{MAX}\;(e_{x,norm}$)	52.1 [38.3, 67.9]	162.7 [138.2, 246.7]	51.2 [38.3, 67.4]	14.8 [10.5, 21.5]

## Data Availability

Data and codes are publicly available through the following open-source repository: https://github.com/H-MOVE-LAB/golf_imu (accessed on 18 May 2026).

## Abbreviations

The following abbreviations are used in this manuscript:

AP	Anteroposterior
CROP	Constrained Redundant Optimization Pipeline
IMU	Inertial measurement unit
MAX	Maximum
ML	Mediolateral
RMS	Root mean square
SO(3)	Special orthogonal group in three dimensions
SP	Stereophotogrammetric
SQP	Sequential quadratic programming
VT	Vertical

## Appendix A

### Appendix A.1. White Noise Propagation in Orientation and Position Estimation

To empirically assess the effect of white noise propagation through the position reconstruction pipeline, a numerical simulation was performed. Accelerometer white noise propagates through the two numerical integrations from acceleration to velocity and from velocity to position, producing an integrated random walk in the reconstructed position. Gyroscope white noise is first integrated during the orientation estimation step and then further propagates through the translational reconstruction, producing a twice-integrated random walk contribution. The aim of this analysis was not to derive a closed form expression for the propagated stochastic error, but rather to quantify its order of magnitude under realistic sensor noise conditions.

The simulated signals had a duration of 5 s and were sampled at 100 Hz. The accelerometer and gyroscope white noise sequences, $n_{a}$ and $n_{g}$, respectively, were generated over three axes using the noise density values reported from the experimental sensor characterization in [37], namely 1.2 mg/√Hz for the accelerometer and 6.5 mdps/√Hz for the gyroscope. These values were converted to discrete time standard deviations by multiplying them by the square root of half the sampling frequency. This resulted in $\sigma_{a}=0.083$ m/s^2^, and $\sigma_{g}=0.046$ deg/s. For each simulation run, three-axis accelerometer noise was added to a static gravity vector, while the three-axis gyroscope noise was generated around zero mean. The resulting noisy IMU signals were processed through the same reconstruction pipeline used in the experimental analysis. First, orientation was estimated using the Madgwick sensor fusion algorithm, with different β values, namely 0 rad/s, 0.001 rad/s, 0.01 rad/s, 0.1 rad/s, and 0.2 rad/s. Then, the accelerometer signal was rotated into the global reference frame, gravity was removed, and the resulting linear acceleration was numerically integrated to obtain velocity and position, according to Equations (10)–(12). The simulation was repeated over 200 independent noise realizations. For each realization, the reconstructed position trajectory was computed along the anteroposterior, mediolateral, and vertical axes. The RMS and maximum absolute position norm error were then calculated over the whole window for each β value and reported in Table A1.

**Table A1:** RMS and maximum absolute position error norm obtained from the white noise propagation simulations for the tested β values, computed over the five second simulation window and reported as median [25th, 75th percentiles] across the 200 simulations.

Position Error Norm (cm)	RMS Median [25th, 75th]	MAX Absolute Median [25th, 75th]
β=0 rad/s	0.4 [0.3, 0.6]	0.9 [0.6, 1.2]
β=0.001$\;\mathrm{rad}/s\;(\beta_{grid}$)	0.2 [0.2, 0.3]	0.5 [0.3, 0.6]
β=0.01 rad/s	0.2 [0.2, 0.3]	0.4 [0.3, 0.6]
β=0.1 rad/s	0.3 [0.2, 0.4]	0.6 [0.4, 0.9]
β=0.2 rad/s	0.5 [0.3, 0.7]	0.9 [0.6, 1.3]

Under the considered noise levels and over the considered simulation interval of five seconds, which is conservative for golf putting analysis, the resulting position error remained limited. Across the 200 simulations and all tested β values, the 75th percentile was below 0.7 cm for the RMS position error and below 1.3 cm for the maximum absolute error along all axes. With $\beta =\beta_{grid}=0.001$ rad/s, the 75th percentile RMS and MAX absolute errors are lower than 0.3 cm and 0.6 cm, respectively. This indicates that white noise has a limited effect on position reconstruction, coherently with the assumptions made in this study. As an example, Figure A1 shows the reconstructed position time series obtained from one of the 200 white noise propagation simulations performed with $\beta =\beta_{grid}$.

### Appendix A.2. Errors Reported for Each Trial

This appendix reports the trial-by-trial reconstruction errors obtained for the 23 analyzed trials. Table A2 presents the orientation, velocity, and position errors obtained from the standard pipeline using $\beta =\beta_{grid}=0.001$ rad/s, whereas Table A3 reports the corresponding errors obtained with CROP. For trial #8, orientation errors could not be computed because of reconstruction issues affecting the relevant stereophotogrammetric marker trajectories. The largest trial maximum error decreased from 1.046 m to 0.426 m. The only exception was trial #10, where CROP produced a slightly larger RMS error, 0.190 m versus 0.141 m, and a nearly identical maximum error, 0.283 m versus 0.281 m.

**Table A2:** Trial-by-trial orientation, velocity, and position reconstruction errors obtained with the standard pipeline using $\beta =\beta_{grid}$. Velocity and position errors are reported along the anteroposterior, AP, mediolateral, ML, and vertical, VT, axes, and as norm-based errors.

	RMS	RMS				MAX	RMS				MAX
Trial	$e_{or}$	$e_{v,AP}$	$e_{v,ML}$	$e_{v,VT}$	$e_{v,norm}$	$e_{v,norm}$	$e_{x,\;\;AP}$	$e_{x,ML}$	$e_{x,VT}$	$e_{x,norm}$	$e_{x,norm}$
**1**	2.1	0.077	0.049	0.174	0.196	0.358	0.063	0.091	0.361	0.378	0.845
2	2.6	0.098	0.022	0.140	0.173	0.332	0.083	0.025	0.246	0.261	0.572
3	2.6	0.065	0.049	0.182	0.200	0.339	0.063	0.071	0.404	0.415	0.935
4	1.6	0.075	0.054	0.169	0.193	0.317	0.059	0.093	0.354	0.371	0.826
5	1.1	0.104	0.022	0.108	0.152	0.324	0.073	0.022	0.148	0.167	0.364
6	1.5	0.081	0.016	0.135	0.158	0.301	0.055	0.015	0.225	0.232	0.512
7	1.1	0.094	0.010	0.104	0.141	0.333	0.079	0.006	0.134	0.156	0.334
8		0.075	0.027	0.126	0.149	0.271	0.067	0.028	0.196	0.209	0.460
9	3.1	0.107	0.050	0.112	0.163	0.361	0.074	0.063	0.154	0.182	0.402
10	2.5	0.161	0.020	0.086	0.184	0.480	0.110	0.015	0.087	0.141	0.281
11	2.9	0.070	0.026	0.137	0.156	0.291	0.029	0.034	0.233	0.238	0.534
12	1.8	0.074	0.048	0.132	0.159	0.317	0.052	0.059	0.222	0.235	0.524
13	1.0	0.112	0.046	0.133	0.180	0.451	0.102	0.024	0.210	0.235	0.513
14	2.3	0.117	0.029	0.085	0.148	0.334	0.065	0.030	0.092	0.116	0.244
15	1.2	0.067	0.029	0.185	0.199	0.331	0.028	0.040	0.408	0.411	0.920
16	3.0	0.133	0.049	0.117	0.184	0.450	0.109	0.060	0.160	0.203	0.456
17	1.5	0.084	0.045	0.134	0.164	0.328	0.044	0.061	0.207	0.220	0.498
18	1.1	0.107	0.024	0.139	0.177	0.440	0.103	0.031	0.228	0.252	0.554
19	1.2	0.072	0.046	0.168	0.189	0.321	0.045	0.067	0.335	0.344	0.776
20	1.2	0.110	0.014	0.101	0.150	0.337	0.077	0.011	0.124	0.147	0.313
21	1.6	0.103	0.023	0.127	0.165	0.371	0.088	0.025	0.199	0.219	0.480
22	1.1	0.057	0.070	0.191	0.211	0.366	0.024	0.143	0.442	0.465	1.046
23	1.3	0.113	0.028	0.104	0.156	0.353	0.082	0.032	0.140	0.166	0.364

Orientation errors are expressed in degrees (deg), velocity errors in meters per second (m/s), and position errors in meters (m).

**Table A3:** Trial-by-trial orientation, velocity, and position reconstruction errors obtained with CROP. Velocity and position errors are reported along the anteroposterior, AP, mediolateral, ML, and vertical, VT, axes, and as norm-based errors.

	RMS	RMS				MAX	RMS				MAX
Trial	$e_{or}$	$e_{v,AP}$	$e_{v,ML}$	$e_{v,VT}$	$e_{v,norm}$	$e_{v,norm}$	$e_{x,\;\;AP}$	$e_{x,ML}$	$e_{x,VT}$	$e_{x,norm}$	$e_{x,norm}$
1	2.3	0.071	0.007	0.008	0.072	0.300	0.042	0.016	0.005	0.045	0.075
2	2.9	0.121	0.018	0.014	0.123	0.350	0.133	0.020	0.012	0.135	0.261
3	2.6	0.070	0.010	0.007	0.071	0.278	0.081	0.019	0.003	0.083	0.122
4	1.7	0.089	0.009	0.012	0.090	0.303	0.113	0.016	0.007	0.115	0.175
5	1.1	0.107	0.009	0.017	0.109	0.350	0.105	0.010	0.009	0.106	0.176
6	1.5	0.089	0.004	0.011	0.090	0.311	0.083	0.004	0.007	0.083	0.150
7	1.0	0.098	0.013	0.009	0.099	0.346	0.090	0.013	0.003	0.091	0.148
8		0.106	0.006	0.009	0.106	0.333	0.164	0.004	0.005	0.164	0.240
9	3.2	0.142	0.010	0.014	0.143	0.459	0.181	0.008	0.006	0.181	0.276
10	3.3	0.173	0.016	0.009	0.174	0.505	0.190	0.014	0.005	0.190	0.283
11	2.8	0.080	0.009	0.020	0.083	0.328	0.079	0.015	0.017	0.082	0.133
12	1.7	0.090	0.025	0.015	0.094	0.354	0.106	0.010	0.008	0.107	0.175
13	0.9	0.111	0.049	0.012	0.122	0.428	0.099	0.035	0.003	0.105	0.190
14	2.4	0.115	0.009	0.016	0.117	0.334	0.070	0.005	0.005	0.070	0.113
15	1.0	0.064	0.006	0.012	0.065	0.302	0.016	0.006	0.017	0.024	0.050
16	2.9	0.103	0.018	0.021	0.107	0.396	0.043	0.015	0.010	0.046	0.069
17	1.4	0.077	0.013	0.024	0.082	0.326	0.022	0.007	0.009	0.025	0.058
18	1.1	0.092	0.019	0.023	0.096	0.411	0.039	0.007	0.013	0.042	0.061
19	1.2	0.082	0.004	0.009	0.082	0.318	0.084	0.007	0.003	0.085	0.134
20	1.2	0.105	0.009	0.011	0.106	0.326	0.077	0.002	0.009	0.077	0.132
21	1.8	0.093	0.007	0.011	0.094	0.327	0.061	0.005	0.005	0.061	0.098
22	3.4	0.204	0.025	0.021	0.206	0.412	0.135	0.045	0.035	0.147	0.426
23	1.4	0.140	0.012	0.028	0.143	0.421	0.135	0.005	0.025	0.137	0.264

Orientation errors are expressed in degrees (deg), velocity errors in meters per second (m/s), and position errors in meters (m).
